# Empowering emerging adults with type 1 diabetes: crafting a financial and health insurance toolkit through community-based participatory action research

**DOI:** 10.1186/s40900-024-00602-1

**Published:** 2024-07-23

**Authors:** Julia E. Blanchette, Claudia B. Lewis, Chantel S. Shannon, Anuhya Kanchibhatla, Jorden Rieke, Mary Jane Roche, Dove-Anna Johnson, Dionne Williams, Shay Webb, Crystal N. Diaz, Erika L. Lundgrin, Nancy A. Allen, Michelle L. Litchman, Betul Hatipoglu

**Affiliations:** 1 Department of Medicine, Diabetes and Metabolic Care Center, Division of Endocrinology, University Hospitals, Cleveland, OH USA; 2 School of Medicine, Case Western Reserve University, Cleveland, OH USA; 3 College of Public Health, Kent State University, Kent, OH USA; 4 Department of Nutrition, Undergraduate Studies, Case Western Reserve University, Cleveland, OH USA; 5 Frances Payne Bolton School of Nursing, Case Western Reserve University, Cleveland, OH USA; 6 The Diabetes Link, Boston, MA USA; 7 College of Pharmacy and Health Sciences, Campbell University, Buies Creek, NC USA; 8 Global Disease Biology, University of California, Davis, CA USA; 9 Divison of Pediatric Endocrinology, Rainbow Babies and Children’s Hospital, Cleveland, OH USA; 10 College of Nursing, University of Utah, Salt Lake City, UT USA; 11 Young Adult Living with Type 1 Diabetes/Lay Person Community Member, New Hampshire, North Carolina, California, USA

**Keywords:** Type 1 diabetes, Health insurance, Financial stress, Health insurance literacy, Emerging adults, Community-engaged participatory action research

## Abstract

**Background:**

Emerging adults aged 18–30 years face challenges during life transitions, with an added burden of navigating the health care system and additional costs associated with diabetes. This stress is compounded by overall low levels of health insurance literacy in this population, as people may not know about available financial and health care resources to minimize suboptimal diabetes outcomes. This study aimed to tailor a financial and health insurance toolkit to emerging adults with type 1 diabetes, including racially, ethnically diverse, and Medicaid-insured individuals, through community-based participatory action research.

**Methods:**

An academic research team and community members from a national organization held six online community advisory board (CAB) content-creation meetings to understand how to tailor a financial and health insurance Toolkit. The CAB was comprised of six racially and insurance-diverse emerging adults with type 1 diabetes and four content experts (clinical, financial, and insurance). Six 60-minute online CAB meetings were held via University Hospitals (UH)-encrypted Zoom over five months. Pre-reading materials were emailed to CAB members before the meetings. A moderator established the purpose of each meeting and briefly discussed meeting rules before each meeting commenced. During the meetings, the moderator guided the discussions and provided the CAB members opportunities to respond and build on one another’s feedback. A deductive thematic qualitative analysis was utilized. Three researchers independently coded the cross-referenced and de-identified CAB meeting transcripts and then convened to reach a group consensus. Two CAB members performed member-checking.

**Results:**

The following key themes emerged to tailor the Toolkit: ensuring that content covers empowerment and self-advocacy, including genuine stories and multimedia visuals for aesthetics, addressing clinician bias, acknowledging racial and ethnic disparities in care, incorporating cultural representation, and demystifying Medicaid stigma.

**Conclusions:**

By successfully partnering with the CAB and a community organization through a community-based participatory action research approach, we will develop a financial and health insurance Toolkit tailored to the needs of racially and ethnically diverse and Medicaid-insured emerging adults with type 1 diabetes.

**Supplementary Information:**

The online version contains supplementary material available at 10.1186/s40900-024-00602-1.

## Abstract (plain language summary)

**Aim of the research:**

This study aims to tailor a financial and health insurance Toolkit to emerging adults, ages 18–30, with type 1 diabetes. Including the insight from racially and ethnically diverse and Medicaid-insured individuals in developing the Toolkit is essential.

**Background to the research:**

Emerging adults with type 1 diabetes have stressful challenges such as navigating the healthcare system, the costs of diabetes, and general diabetes self-management. This stress is worsened by low levels of health insurance literacy and leads to suboptimal diabetes outcomes. This issue affects many individuals but dramatically impacts those who are racially and ethnically diverse or Medicaid-insured.

**Design and methods used:**

Six online content-creation meetings were held to understand the Toolkit content needs and preferences. We analyzed the meeting transcripts to uncover common themes. Patient and public involvement: An academic research team, a national organization (The Diabetes Link), and a Community Advisory Board (CAB) partnered together. The CAB members were racially and insurance-diverse emerging adults with type 1 diabetes and content (financial, insurance, clinical diabetes) experts. We will continue to collaborate with the CAB members to develop a research protocol to test the effects of the Toolkit.

**Dissemination:**

The research findings will be shared with young adult type 1 diabetes stakeholders, healthcare providers, and community and professional organizations. Dissemination strategies will include publications, community and scientific conference presentations, community events, and social media resources and content. The finalized Toolkit will be publicly available on the Diabetes Link Resource Hub.

**Supplementary Information:**

The online version contains supplementary material available at 10.1186/s40900-024-00602-1.

## Background

Emerging adults aged 18 to 30 years with type 1 diabetes are a high-risk group for detrimental self-management outcomes, as only 14% meet the optimal glycemic target of A1c < 7.0% [1, 2]. Emerging adulthood is a critical life stage of many transitions, including from pediatric to adult healthcare environments, navigating relationships, pursuing higher education, starting a career, balancing financial instability, and learning new coping skills [3]. Emerging adults with type 1 diabetes experience these challenges while learning to navigate the complexities of the healthcare system, health insurance, and health care finances [3–5]. However, health care transition and self-management are further complicated by financial and emotional instability, career changes, and low health insurance literacy (HIL) [3, 6–8].

Health insurance literacy is the degree to which an individual has the knowledge, ability, and confidence to find and evaluate information about private and public health insurance plans and to select, enroll, and utilize the best plan for their financial and health circumstances [9]. Uninsured emerging adults with low HIL have a heightened risk of hospitalization for hyperglycemia [10]. Even for the insured, the emotional stress of costly diabetes care and low HIL both negatively impact A1c, insurance coverage, diabetes care, and out-of-pocket costs [11–16].

Financial and health insurance resources are available to the general population [17] and college-aged students [18], but these resources are not tailored to meet the learning and literacy needs of emerging adults with chronic conditions such as type 1 diabetes. Emerging adults frequently engage with micro video-based social media content (e.g., TikTok, Snapchat) tailored to their learning preferences [19, 20]. Online media platforms are ideal avenues for short, simple videos on various insurance topics that have been shown to increase health literacy [21, 22]. A cornerstone of engaging in impactful diabetes self-management education is incorporating peer support and social networks, which can be achieved through online learning content featuring peers [23, 24]. Therefore, we previously developed and pilot-tested a type 1 diabetes Financial Toolkit (pilot Toolkit) [25] that highlighted peers living with type 1 diabetes in an online micro video series to support the digital native learning style of this population [26].

## Summary of previous work

**Fig. 1:**
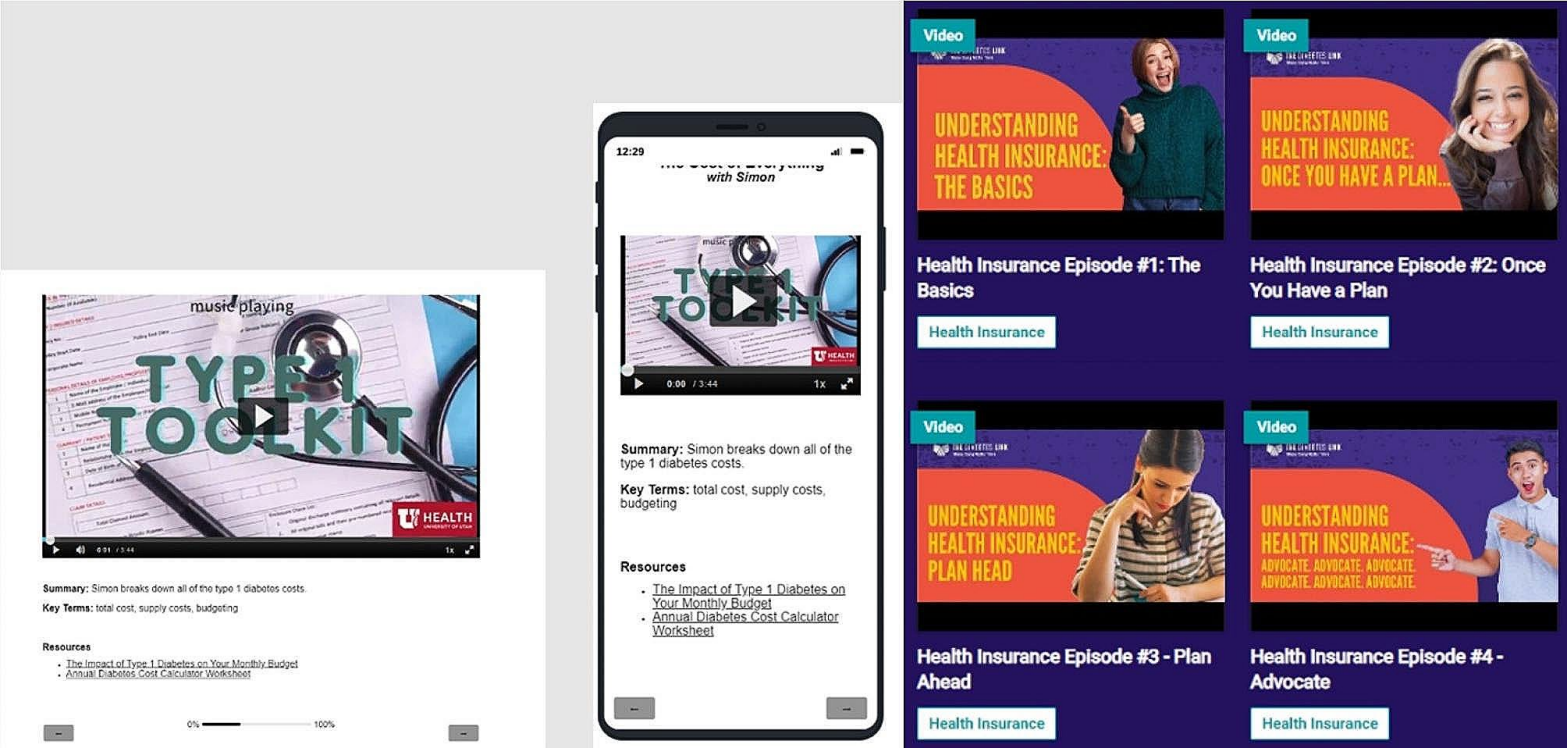
Pilot Work: Toolkit on web browser and phone (Left) and diabetes link resource hub (Right)

Our pilot study involved using a community advisory board (CAB) to assist in developing the pilot Toolkit (Appendix A). The pilot Toolkit was developed using a systematic and iterative process with *N* = 6 CAB members of various backgrounds representing emerging adults aged 18 to 25 years living with type 1 diabetes. The average age of the CAB was 38.5 years; nearly 67% were female, approximately 83% were White, 17% were Asian, and 33% were Hispanic. There were *n* = 2 emerging adults with type 1 diabetes. Other relevant persons involved in the CAB included nonprofit community partners (a parent of an emerging adult with type 1 diabetes), a double-board certified pediatric and adult endocrinologist, a diabetes care and educational specialist, and a financial educator (a parent of an emerging adult with type 1 diabetes). The pilot Toolkit videos featured four emerging adults living with type 1 diabetes from diverse racial, ethnic, geographic, and career backgrounds. The content areas the CAB selected included enrolling in health insurance plans, distinguishing between plans featuring health savings accounts (HSAs) versus flexible spending accounts (FSAs), health insurance plan options and qualifications, careers that offer health insurance, and budgeting for diabetes care costs (Fig. 1, Left).

The pilot Toolkit (Appendix A) was feasible and acceptable and had a 100% completion rate in a 30-day window [25]. All participants in the pilot study were satisfied and found the Toolkit easy to navigate, engaging, and understandable [25]. However, the pilot Toolkit was developed for and tested in highly educated, privately insured, White, non-Hispanic individuals [25]. Additionally, we set a cutoff age of 25 for the pilot participants because we aimed to target emerging adults before they became insurance policy carriers, yet 33% of the pilot study participants were already insurance policy carriers or were on a spouse’s policy. To better represent the diversity of this population, the research team recognized that the pilot Toolkit needs to be modified and tested in emerging adults beyond age 25 from various backgrounds, races, ethnicities, and socioeconomic levels. Tailoring health communication and interventions to the target population they are intended to serve is an efficient and cost-effective method for promoting and sustaining health behavior change [27]. For example, tailored text-messaging interventions for type 2 diabetes management contributed to improved glycemic outcomes [28] and culturally tailored diabetes self-management education and support demonstrated the potential to improve psychological health among Hispanic, Latino, and African-American persons with diabetes [29]. These previous studies highlight the importance of modifying the pilot Toolkit to be tailored to the larger population of emerging adults with type 1 diabetes.

To ensure that the Toolkit will be a sustainable resource that is widely disseminated, the academic research team collaborated with a community organization, The Diabetes Link, for the next phase of Toolkit development. The Diabetes Link is a national nonprofit organization with the mission of empowering young adults with diabetes to thrive by providing access to peer support and resources. As the pilot Toolkit data were being collected, the Diabetes Link was separately testing the user engagement of an independently developed Insurance 101 Video Series on its new young adult resource hub. The Resource Hub is a collection of online resources used to help address the issues that emerging adults most commonly face. The videos had more than 28,000 social media shares, saves, likes, and views (Fig. 1, Right).

## Methods

### Aims

Phase one of the current study aims to develop a Type 1 Diabetes Financial and Health Insurance Toolkit by adapting the pilot Toolkit and Insurance 101 videos to the needs of a broader population under the guidance of a CAB. Our primary research questions for the CAB were as follows:

1. What are the key content areas to include in the Toolkit?
2. What should the Toolkit look like?
3. How can we tailor the Toolkit to racially and ethnically diverse individuals?
4. How can we tailor the Toolkit to those on Medicaid?

Community-based participatory action research (CBPR) was utilized to develop, implement, and disseminate a Toolkit to meet the financial and health insurance needs of emerging adults with type 1 diabetes across racially and ethnically diverse and publicly insured communities. Importantly, we aimed to ensure that no research practices or interventional educational materials developed contributed to the stigmatization of historically marginalized communities [30]. Therefore, the aim of patient involvement was to integrate community partners and members throughout the intervention development and research process by including the Diabetes Link staff and a CAB composed of emerging adults with type 1 diabetes and financial and insurance content experts. The CAB feedback was used to design the online Financial and Health Insurance Toolkit to serve this unique population in improving finances, HIL, and self-management outcomes.

### Recruitment

Purposive recruitment of CAB participants was done via a diabetes nonprofit community organization, The Diabetes Link, and a diabetes clinic at a large Midwestern academic medical center per provider recommendation to ensure racial, ethnic, gender, and insurance diversity. We recruited racial and ethnically diverse participants with a variety of insurance types, including Medicaid, to incorporate the perspectives of these community members. As the Toolkit will be an educational financial and insurance resource, we included content experts in the following roles: insurance educators, financial educators, diabetes care and education specialists, and endocrinologists. Content experts were recruited from two different academic medical centers and through the Diabetes Link. The content experts previously volunteered with the academic medical centers or The Diabetes Link community events. Table 1 shows the CAB member roles.

**Table 1:** Community advisory board member roles

Targeted CAB roles	Filled CAB roles
Emerging adults	
Racially/ethnically diverse emerging adult with type 1 diabetes	Racially/ethnically diverse emerging adult with type 1 diabetes, on private insurance
Racially/ethnically diverse emerging adult with type 1 diabetes	Racially/ethnically diverse emerging adult with type 1 diabetes, on private insurance
Racially/ethnically diverse emerging adult with type 1 diabetes	Racially/ethnically diverse emerging adult with type 1 diabetes, on Medicaid, previously uninsured
Racially/ethnically diverse emerging adult with type 1 diabetes	Racially/ethnically diverse emerging adult with type 1 diabetes, on Medicaid
Medicaid-insured emerging adult with type 1 diabetes	Medicaid-insured emerging adult with type 1 diabetes
Marketplace-insured emerging adult with type 1 diabetes	Marketplace-insured emerging adult with type 1 diabetes
**Content Experts**	
Insurance educator	Insurance educator
Financial educator	Financial educator, person with type 1 diabetes
Young adult endocrinologist	Young adult endocrinologist, person with type 1 diabetes
Certified Diabetes Care and Education Specialist (CDCES)	CDCES, person with T1D, parent of an emerging adult with type 1 diabetes

### Methodology

Over five months (June-October 2023), six 60-minute online CAB meetings were held via University Hospitals (UH)-encrypted Zoom sessions. Zoom CAB meeting groups are cost-effective, user-friendly, convenient, and helpful in establishing and maintaining rapport [31]. Virtual Zoom meetings were determined to be appropriate for all CAB members because they were not naïve to the Zoom platform, had appropriate hardware to attend the meeting, and had high-speed internet connections [32].

At least one week before each meeting, the research coordinator (DW) emailed the Zoom invitations and pre-reading materials to the CAB. The pre-reading materials included the pilot Toolkit (ten videos, three minutes and 30 s on average each) and the Health Insurance 101 videos from The Diabetes Link (three videos, four to seven minutes each). The CAB was instructed to read the materials and proactively formulate feedback to enhance the meeting flow and pace of conversation. Brevity was a priority in creating pre-reading materials to reduce burden and enhance retention of the CAB. Text messages and 48-hour reminder emails were also used to enhance meeting attendance. The research coordinator (DW) and the Diabetes Link team (MJR) were available to answer questions before the CAB meetings and via email. Absentee participant forms were extended to members who could not attend live meetings to allow for an opportunity to provide feedback.

To the research team’s knowledge, there is little research about conducting online CAB meetings. Therefore, the research team followed best practices for online focus groups. Zoom focus groups are cost-effective, user-friendly, convenient, and useful for establishing and maintaining rapport [31]. A research investigator (JEB) served as the CAB moderator. At the start of the focus group sessions, the moderator established the purpose of the meeting, CAB member roles, and “ground rules,” such as muting when not speaking and using the chat feature instead of interrupting others. The CAB gave introductions (first names only) and participated in an icebreaker before the sessions began. Pre-reading materials provided to the CAB members before focus group sessions and semi-structured interview guide questions during the sessions helped guide the focus and flow of conversations and ensured all vital content areas were discussed. Table 2 displays each CAB focus group session’s goals and the questions asked during each session. The CAB members were compensated for their time for each session attended, $100 per hour of work provided by the academic medical center conducting the research with allocated research grant funding.

**Table 2:** CAB sessions and semi-structured interview questions

CAB session	Session goal	Interview questions
**1**	Review the current Toolkit as a group and discuss each video.	**RQ1. What are the key content areas to include in the Toolkit?** A. What video content should be removed or re-filmed? B. What video content is essential to keep? C. What content do we need to add?
**2**	Tailoring the Toolkit (aesthetic of current videos, culture of current videos, content to add or remove from current videos)	**RQ2. What should the Toolkit look like?** **RQ3. How can we tailor the Toolkit to racially and ethnically diverse individuals?** **RQ4. How can we tailor the Toolkit to those on Medicaid?**
**3**	Propose New Toolkit Content Areas and Begin to Refine Content for Video Production Team	1. Please review the new content titles and their order. How can the titles be rearranged to tell a better story? 2. Based on your feedback, we plan to weave self-advocacy into as many videos as possible. What are your thoughts on this? Please review content areas 1, 2, and 3. 1. What do you think the official title should be? 2. What feedback do you have about the content? 3. What should be changed, if anything, from the goals, objectives, and learning outcomes? 4. What do you think of the resources/sources for this video? Should anything be added or removed?
**4**	Refine Content for the Video Production Team	Please review content areas 4, 5, and 6. 1. What do you think the official title should be? 2. What feedback do you have about the content? 3. What should be changed, if anything, from the goals, objectives, and learning outcomes? 4. What do you think of the resources/sources for this video? Should anything be added or removed?
**5**	Refine Content for the Video Production Team	Please review content areas 7, 8, 9, and 10. 1. What do you think the official title should be? 2. What feedback do you have about the content? 3. What should be changed, if anything, from the goals, objectives, and learning outcomes? 4. What do you think of the resources/sources for this video? Should anything be added or removed?
**6**	Refine Content for the Video Production Team	Please review content areas 11, 12, and 13. 1. What do you think the official title should be? 2. What feedback do you have about the content? 3. What should be changed, if anything, from the goals, objectives, and learning outcomes? 4. What do you think of the resources/sources for this video? Should anything be added or removed?
*Over six months, the research team (Principal investigator, a clinical diabetes expert) and community partner (young adult learning experts from The Diabetes Link) team met with the video production company to discuss each content area Face Sheet (a one-page content summary for each content area) in detail, review the CAB recommendations, evaluate the video content for accuracy and ease of understanding, and recruit a diverse group of emerging adults with T1D and healthcare providers to be featured in the video series.*		
**Future: 7**	Review revisions as a group. Watch any videos with revisions and confirm acceptability. Additional modifications and confirmation of acceptability will be made until all CAB members approve the Toolkit as acceptable.	

### Data management

The CAB members self-reported their demographics via a REDCap survey. The CAB meetings were audio recorded, and chat text and audio transcripts were produced for each meeting via encrypted Zoom recording features. The absentee forms included the same CAB meeting questions; text responses were combined with the Zoom transcripts. Combined transcripts were organized and de-identified for subsequent analysis. De-identification included removing member names, workplace names, and any other potential identifiers to ensure privacy and confidentiality.

### Data analysis

Deductive thematic analysis was conducted with qualitative rigor and met the steps of trustworthiness—credibility, transferability, and confirmability [33]. All transcripts were de-identified and cross-referenced by three research team members in alignment with thematic analysis and data confidentiality best practices before being uploaded to NVivo version 14 for analysis [34]. Research team members independently applied a priori codes from the research questions to identify recurring themes. Codes were expanded or collapsed as appropriate to define, organize, and describe themes clearly. Additional codes highlighted pertinent information that did not fall under specific categories. To minimize confirmation bias, a fourth team member, researcher, and investigator who did not code cross-checked and refined the codes to reach a consensus. Two patient CAB members (SW and CND) conducted member-checking by reviewing the analysis findings to ensure data credibility [35]. Two of the patient CAB members also served as co-authors of this paper. The GRIPP2 Short Form Checklist was utilized throughout manuscript preparation to report patient involvement in this research [36].

## Results

### Demographics

The CAB was comprised of ten members, with 100% retention, defined as participation or absentee form completion for all six CAB sessions. Gender was represented by six females, three males, and one non-binary member. All members had a bachelor’s degree or higher education. For descriptive purposes, the CAB was split into content experts (*n* = 4; diabetes care and education specialist; young adult endocrinologist; financial content expert; and a health insurance content expert) and emerging adults (*n* = 6). The mean (SD) age was 50.75 (7.04) years among the content experts and 26.6 (7.26) years among the emerging adult members. Among the emerging adult members, the races represented included Black (*n* = 2), Asian (*n* = 2), and White (*n* = 2). The health insurance types of the emerging adult members included Medicaid (*n* = 3, 50%), private employer-sponsored (*n* = 2, 33%), and marketplace (*n* = 1, 17%).

### Semi-structured CAB group interviews

**Fig. 2:**
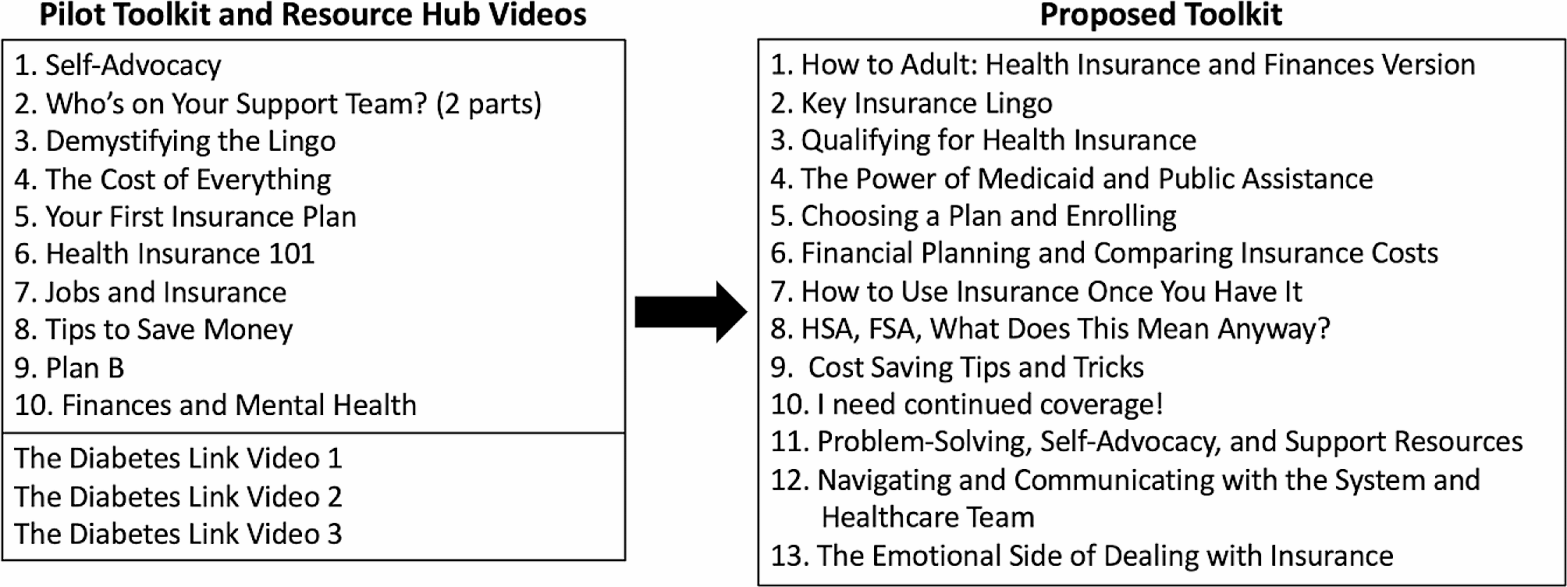
Pilot toolkit and proposed toolkit content

The CAB identified and approved thirteen content areas that informed modifications to the pilot Toolkit (Appendix A) structure and content (Fig. 2). See Appendix B for an overview of the revised Toolkit.

Revising the ten pilot Toolkit and Diabetes Link Insurance 101 videos involved creating, reviewing, and approving video face sheets. Video face sheets were created via collaborative efforts by research team members (JR, CS, JB) and the Diabetes Link staff (MJR & KS). Themes identified from the deductive thematic analysis, insights gained from notes taken during the CAB group interviews, and content areas described by CAB members were utilized to create the face sheets. A face sheet was created for each revised video and included the proposed video title, themes to cover within the video, a video overview and description, and what goals, objectives, and learning outcomes viewers should be able to meet after watching the video. Learning outcomes were developed using a hierarchical model called Bloom’s Taxonomy, which categorizes learning in the order of reproduction, understanding, application, analysis, evaluation, and creation [37]. Learning outcomes associated with each module can be found in Appendix B. The face sheets also included insights to ensure the emerging adult lens, or thoughts, behaviors, feelings, and experiences emerging adults tend to experience, were represented appropriately in each video. In addition, the face sheets contained recommendations for volunteers or speakers to be in the video and what attributes (e.g., race, ethnicity, gender, age, tone of voice, general disposition) they should have. Lastly, a content outline was included in each face sheet that detailed what resources would be utilized to source the information for the video, what resources needed to be developed (e.g., a downloadable Excel file of a budgeting spreadsheet needed to be developed for video six: Financial Planning and Comparing Insurance Costs), and research team contacts.

Face sheets were reviewed by the research team (JR, CS, JB) and Diabetes Link staff (MJR & KS) for accuracy, clarity, and completion before they were sent to CAB members for additional review and feedback. CAB members were encouraged to provide feedback on any aspect of the proposed revised video that they felt needed to be changed, edited, removed, or added. The third, fourth, and sixth CAB group interview sessions focused on discussing CAB members’ feedback on the video face sheets. The fifth CAB group interview session was not held on Zoom due to conflicting schedules for most CAB members. Instead, CAB members were instructed to fill out a CAB meeting assignment sheet with their feedback and email the sheets back to the research team for subsequent review. Face sheets were reviewed, and feedback was discussed in batches of three.

During the third, fourth, and sixth CAB group interview sessions, CAB members were asked the following questions for each video face sheet and permitted time to elaborate on their responses:

1. What do you think the official title should be?
2. What feedback do you have about the content?
3. What should be changed, if anything, from the goals, objectives, and learning outcomes?
4. What do you think of the resources/sources for this video? Should anything be added or removed?

Themes from the qualitative analysis are organized by research questions and exemplary quotes provided by patient CAB members.

### Research question 1. What are the key content areas to include in the revised Toolkit?

Two primary themes emerged for the revised Toolkit: empowerment and self-advocacy content.

### Empowerment

CAB members emphasized the importance of incorporating empowerment throughout all key content areas. Empowerment was especially highlighted during discussions about career planning and problem-solving. The CAB noted that emerging adults should pursue their desired careers and not allow diabetes to dictate their future. The importance of supporting emerging adults with type 1 diabetes in following their dreams can be exemplified by the following quote from a financial and insurance expert CAB member:

*“That broke my heart [referring to a pilot Toolkit video portraying a young adult picking a career in education over performing arts for insurance purposes]… Are we trying to get people to change their lives and a career? If I was an advocate, I want that person to have the career they want to have. We should be making sure that the [insurance] exchanges give them the opportunity to buy the insurance that they want.”*

### Self-advocacy

Self-advocacy was also part of discussions across content areas. The CAB members highlighted that asking questions at each stage of navigating the health care system was a simple, effective self-advocacy strategy that emerging adults could use. One financial and insurance expert CAB member explained how emerging adults must not give up, regardless of the situation or environment, because barriers can occur at any stage of navigating the health care system:

*“My number one thing that I would add to that video is, don’t ever give up… If you get a denial, don’t give up. The insurance company expects you to give up. Don’t give up… anywhere you’re in a healthcare setting, you’re advocating for yourself.”*

### Research question 2. What should the Toolkit look like?

The two primary themes that emerged for Toolkit aesthetics included multimedia visuals and genuine stories.

### Multimedia visuals

CAB members emphasized the need to enhance the videos in the Toolkit with graphics, overlaid text, and video transcripts. One CAB member explained how graphics could illustrate the content to emphasize its delivery:

*“I really enjoyed the different graphics…. I’m pondering, and this is my thinking face … they actually really help with that.”*

The CAB shared that reading transcripts while watching television or multitasking when listening to a video’s audio is common. Including transcripts in the Toolkit will also accommodate learning styles and enhance accessibility. As one emerging adult CAB member shared,

*“I really enjoy having the transcript to the side so I can kind of read along and listen… cause like, I know there are a lot of different types of learners out there, so it might be helpful.”*

### Genuine stories

CAB members routinely used words such as ‘genuine,’ ‘human,’ ‘real,’ ‘relatable,’ ‘personal,’ and ‘stories’ to describe the suggested tone or feel for the Toolkit videos. They felt genuine stories and personal narratives were critical to engaging viewers and successfully communicating video content. Some statements to illustrate this point include the following:

*“Realism is key, personal stories that people can relate to.” “Genuine = vulnerability sharing experiences.” “Genuine to me means human. Like, you can tell it’s not just someone staring at a screen and reading a script.”*

### Research question 3. How can we tailor the Toolkit to racially and ethnically diverse individuals?

Three themes emerged to tailor the Toolkit to racially and ethnically diverse individuals: addressing clinician bias, acknowledging health disparities, and representing diverse cultures.

### Addressing clinician bias

CAB members identified the need for the Toolkit to address clinician biases that could impact type 1 diabetes care. One emerging adult CAB member described how biases toward patient characteristics can cause harm when she said,

*"Think about identifying folks that you cannot advocate to, whether that be like a doctor who’s particularly fat phobic… a nurse who’s particularly racist… somebody who doesn’t want to prescribe these certain medicines, or makes it really hard for you to get on a CGM because, you know, they want you to get used to finger pricks first…and maybe the best form self-advocacy is finding another source to get that care."*

Another emerging adult CAB member underscored how misperceptions about the actual costs of type 1 diabetes could prevent meaningful conversations about insurance coverage and financial planning:

“*But I think it would be interesting to have a video that addressed ableism… people think like, okay, certain people make this salary, or they go to this school—obviously they can afford this. But they don’t know the breakdown of how much diabetes really costs.”*

### Acknowledging health disparities

CAB members discussed health disparities across content areas. One emerging adult CAB member expanded the discussion to highlight the far-reaching nature of health disparities, which are not limited to racial or ethnic identity but include comorbid conditions and disability:

*”And then on top of that, being a person who’s comorbid and has other things other than diabetes, how that factors into it is a huge thing.”*

### Cultural representation

CAB members identified cultural representation as an essential consideration for aesthetics and content in the Toolkit. Cultural influences impact financial opportunities and decision-making. One emerging adult CAB member stated,

*“I’m from an Asian background, and I think a lot of the financial things are influenced by my family. So, kind of including examples surrounding that. Like how to deal with it when you’re also talking with your family too… So kind of including that cultural influence into it as well.”* The discussion was summarized with the simple statement, *“Representation is important.”*

### Research question 4. How can we tailor the Toolkit to those on Medicaid?

The key theme that emerged from the discussion was “demystifying Medicaid.”

#### Demystifying medicaid

CAB members underscored the need to frame Medicaid as a positive, helpful resource and to oppose the stigmatization that dissuades individuals from utilizing it. The insurance expert CAB member shared,

*“We have to talk about that this is your right to have that Medicaid… and paint it in a better picture than I think a lot of people are feeling about it.”*

One emerging adult CAB member illustrated the benefit of Medicaid for type 1 diabetes care:

*“My insulin was actually cheaper when I was on Medicaid and I qualified for so many more coupons from the manufacturers, there’s a lot of great perks.”*

Similarly, another emerging adult CAB member lamented not taking advantage of Medicaid resources when eligible:

*“I didn’t know I was eligible for Medicaid and stayed on my parents’ high deductible plan. When in reality I was not dependent on my parents anymore, living in a different state, a grad student not making any money, and could have been enrolled in Medicaid the whole time.”*

## Discussion

Over five months, an academic research team and The Diabetes Link, a nonprofit organization dedicated to helping emerging adults with diabetes thrive, used a CBPR approach and collected qualitative information to enhance a Financial and Health Insurance Toolkit for emerging adults with type 1 diabetes. During CAB meetings, six type 1 diabetes community members and four content area experts were invited to provide a range of perspectives and guidance on addressing racial, ethnic, and insurance plan diversity; cultural competency; and content appropriateness to enhance and develop this Toolkit. We were impressed by the CAB retention, member engagement, and dedication to improving the community’s finances and HIL.

The first research question investigated content changes to the pilot Toolkit and found that empowerment and self-advocacy were key messages to relay. The empowerment of patients has been part of health care for decades through informed consent, yet emphasizing empowerment during financial health education has been rare [38]. Providing emerging adults with type 1 diabetes tools and resources to increase their confidence in understanding and utilizing their health insurance benefits can empower them to feel secure during life transitions and achieve their self-management goals. Self-advocacy is a product of empowerment. Once emerging adults understand their health insurance benefits, they can better navigate health care and achieve their goals [39]. Tenacity is essential for self-advocacy, as perseverance is required when contacting insurance agents or health care providers for days, weeks, or months to obtain the care one needs.

Furthermore, the CAB noted that Toolkit videos should be inviting and inclusive and recommended using anecdotes and personal stories. The Social Learning Theory explains that individuals learn by observing modeled behaviors and imitating their attitudes and responses [40]. Emerging adults watch others living with chronic conditions on social media to gain knowledge about self-management for daily lived experiences [41]. Adding conversation-style videos and realistic stories will allow viewers to develop a sense of partnership with resources while fostering a friendly learning environment.

Our third research question revealed that the Toolkit could be tailored for racially and ethnically diverse individuals by addressing clinician bias, acknowledging health disparities, and representing diverse cultures. Clinician bias is intimidating for emerging adults, creates unsafe spaces, and has been found to worsen equity gaps in the use of diabetes technology [42]. Like previous research findings, emerging adult CAB members commonly experienced insurance-mediated provider bias when on public insurance and general bias, including A1c threshold requirements for prescriptions [42–45]. Acknowledging these disparities while providing emerging adults with the knowledge and resources to navigate provider bias is essential in building diabetes self-management skills. Thus, these messages are critical for inclusion in the Toolkit.

Additionally, CAB members emphasized the impact of family culture on diabetes finances. Individuals’ beliefs and cultural beliefs about risk-taking, savings, and reliance on families affect their financial decision-making [46]. Navigating these new responsibilities as an emerging adult can be overwhelming. To address this concern, we will highlight the diversity of representation throughout all the videos to share genuine stories that resonate with various cultural backgrounds.

The primary theme that emerged from the discussion of how to tailor the Toolkit to individuals on Medicaid was to demystify Medicaid. Public health insurance programs such as Medicaid are invaluable for helping eligible emerging adults obtain the diabetes supplies, medications, and care required to thrive. Like other public assistance programs, Medicaid is shrouded in stigma, which may deter emerging adults from enrolling [47]. Emerging adults generally have low knowledge of health insurance plan types, contributing to potential Medicaid misconceptions [6] and the temporary loss of Medicaid coverage, known as churning [48, 49]. Previous studies have shown that younger adults with chronic conditions fear being treated differently by health care clinicians when enrolled in Medicaid [47]. The Toolkit will provide an approachable introduction to Medicaid to combat stigma and address Medicaid misconceptions.

Previous and current evidence supports that collaborating with CABs to inform health research and interventions is advantageous in developing research questions and interventions important to the community or target population [50, 51] and increasing community members’ access to empowering research tools and results [52–54]. Dissemination of health education resources and interventions is an essential function of health promotion, as it spreads knowledge, evidence-based interventions, and the associated benefits of increased knowledge or resulting behavior change [55]. CABs involvement in health research and the development of health interventions yield valuable insight into tailoring health messaging [56], an essential communication technique for successfully disseminating public health interventions and uptake in target populations [55], further underscoring CAB’s importance to public health and health promotion.

Historically, emerging adults experience the highest rates of being uninsured or underinsured, implying that they are in need of additional support to navigate the transition [4]. In order to make it a smoother transition, American Diabetes Association consensus recommendations encourage providers to educate patients on strategies to maintain health insurance coverage and understand what coverage options are available to them [4]. One way to communicate that information is by meeting emerging adults where they are, like providing internet-based resources [38]. The use of online communication tools are widely used by emerging adults because of their accessibility [21]. For example, disseminating HIL information via social media outlets like Instagram and YouTube would provide more opportunities to consider health insurance outside of the clinic. Emerging adults can also better comprehend HIL materials, such as short videos, when presented in a way that aligns with learning preference [21, 25]. Existing literature suggests that materials should combine creative, engaging, and quality content to communicate health insurance information most effectively [21]. The Toolkit addresses these needs by providing resources that are easily accessible online for emerging adults and their health care team and, in the future, disseminated via social media.

### Limitations

Our study has limitations. Although the CAB represented racial, social, and financial diversity, no member self-identified as Hispanic. However, the pilot Toolkit CAB had two Hispanic members (33.3%), and only 30.8% of participants in the pilot study were Hispanic [25]. To ensure that the modified Toolkit will be applied to Hispanic individuals, we will feature Hispanic emerging adults in the videos and request feedback from Hispanic individuals with type 1 diabetes. Furthermore, all CAB members had at least a bachelor’s degree, underrepresenting those who had completed high school, trade school, or some college. Additional representation gaps included those with diverse family dynamics, such as having children. To address these gaps, the Diabetes Link will collaborate with emerging adults who fit these demographics for Toolkit review and will feature their voices in the videos. Future phases of this collaboration will test the Toolkit nationwide in racially and ethnically diverse (at least 50%) and Medicaid-insured (at least 50%) emerging adults aged 18–30 years. We also aim to include individuals with varying education levels by recruiting from clinics that serve under-resourced communities. In continued collaboration with the CAB, we will develop a randomized controlled trial protocol, update the Toolkit based on their feedback, and disseminate the Toolkit to the type 1 diabetes community.

## Conclusion

Through CBPR, collaborative efforts among an academic research team, a community organization (The Diabetes Link), and a CAB compiled recommendations for creating an enhanced Financial and Health Insurance Toolkit for emerging adults with type 1 diabetes. To our knowledge, this will be the only resource explicitly tailored to this population. The CAB was pivotal in shaping the Toolkit’s content and approach, as it recommended that it address empowerment and self-advocacy through genuine stories and multimedia visuals. Notably, the CAB suggested that addressing clinician bias, acknowledging health disparities, and representing various cultures would make the Toolkit relevant to racially and ethnically diverse individuals. The CAB also advocated for empowering emerging adults by demystifying Medicaid to combat its associated stigma. In the subsequent phases of our partnership, we will test the Toolkit in a randomized controlled trial. In the long term, we aim to disseminate a sustainable, engaging, and accessible financial and health insurance Toolkit via a community-led online resource hub. This collaborative approach underscores the importance of CBPR in developing valuable resources to empower and support emerging adults with type 1 diabetes in managing their health and insurance needs.

### Electronic supplementary material

Below is the link to the electronic supplementary material.

Supplementary Material 1

Supplementary Material 2

Supplementary Material 3

## Data Availability

No datasets were generated or analysed during the current study.

## Abbreviations

CAB: Community advisory board
CBPR: Community-engaged participatory action research
HIL: Health insurance literacy
