# Supplementary material for: Empowering emerging adults with type 1 diabetes: crafting a financial and health insurance toolkit through community-based participatory action research
Source: Res Involv Engagem. 2024 Jul 23;10:75. doi: 10.1186/s40900-024-00602-1 (PMC11265338; doi:10.1186/s40900-024-00602-1)
Supplement: Supplementary file 1 — Supplementary Material 1 [file 40900_2024_602_MOESM1_ESM.docx]

**Appendix A. Pilot Toolkit Overview**

| **Video Title** | **Length (minutes)** | **Description** |
| --- | --- | --- |
| 1. Self-Advocacy | 3:30 | **Summary:** AK shares encouragement and tips to help you advocate during insulin, supply, health insurance, or financial barriers. She discusses self-advocacy and problem solving when working with your insurance provider, through insurance paperwork and prior authorizations.  **Online Resources Provided:** Denials / Appeals: What to Do When Your Insurance Company Denies Your Coverage (JDRF), When Insurance Gets Turned Down: Appeals Explained (Diatribe), Insurance Company Switch Your Medication? Fight Back (Diatribe) |
| 2. Who’s on Your Team | 2:24  3:16 | **Summary**: AK speaks about personal, stressful experiences with T1D care, and how to identify people on your support team (emotional support and healthcare support) to help you navigate these barriers with resources such as care coordinators.  **Online Resources Provided:** The Friends & Family Guide to Type 1 Diabetes (Beyond Type 1), The Boyfriend/Girlfriend Guide to Caring for Someone with Type 1 (Beyond Type 1) |
| 3. Demystifying the Lingo | 2:51 | **Summary:** SW introduces, demystifies and defines commonly used health insurance terms (premium, deductibles, co-insurance, copay).  **Online Resources Provided:** Common Health Insurance Terminology 101 (JDRF) |
| 4. The Cost of Everything | 3:44 | **Summary:** SW breaks down all of the T1D costs including insurance premiums, deductibles, co-insurance, and copays, and helps you think through identifying your costs and budgeting.  **Online Resources Provided:** At What Cost? The Impact of Diabetes on Your Budget, Part 1 (Beyond Type 1), 10 Steps to Understanding Your Annual Healthcare Costs (Affordable Insulin Project) |
| 5. Your First Insurance Plan | 4:17 | **Summary:** JA shares her experiences and helpful tips for when you enroll in your first health insurance plan and are in charge of your healthcare and bills. She reviews choosing an insurance plan, associated paperwork and bills, receipt and bill organization, switching pharmacies, and scheduling flexible appointments, such as via telehealth.  **Online Resources Provided:** Health Insurance Guide for T1D Navigation Guide (Beyond Type 1), Aging Out of Parent’s Insurance: What Are Your Options? (Diatribe) |
| 6. Health Insurance 101 | 3:34 | **Summary:** JA introduces and explains the different types of health insurance plans (public insurance/Medicaid, employer-sponsored private insurance, health insurance marketplace) and requirements to enroll in them. She also discusses key components of the different plans (example: where you can get healthcare services if you have a Preferred Provider Organization versus Health Maintenance Organization plan) and provides additional tips such as understanding health savings accounts and flexible spending accounts.  **Online Resource Links:** How to Choose the Best Health Insurance Plan for People with Type 1 Diabetes: Key Questions to Ask (JDRF), The Deductible Problem (Beyond Type 1), Finding Health Insurance (usa.gov) |
| 7. Jobs and Insurance | 3:59 | **Summary:** MR introduces types of careers, including union jobs and part-time jobs with benefits, and jobs to consider when wanting or needing health insurance benefits.  **Online Resource Links:** Skip the Coverage Gap: Maintaining Diabetes Care While Changing Insurance (Diatribe), 7 Employers That Offer Part-Time Employees Health Insurance (MoneyUnder30.com) |
| 8. Tips to Save Money | 3:49 | **Summary:** JA shares tips to save on insulin and diabetes supply costs. Examples include ordering 90-day prescriptions (if available), available coupons, and utilizing pharmacy benefits for some diabetes supplies (if available).  **Online Resource Links:**  10 Ways to Save Money on Diabetes Care (Diatribe), How to Save Money on Diabetes Medications with Prescription Discount Cards (Diatribe), When and Why to Consider Mail-Order Prescriptions (Diatribe) |
| 9. Plan B | 4:53 | **Summary:** SW shares options to access insulin and supplies when you can't afford them. Examples include what to do when you need a prior authorization, have an expired prescription or no refills, can’t afford the out-of-pocket costs and programs such as state copay caps, patient assistance programs, and emergency options such as discussing over-the-counter generic insulin with your diabetes clinician, acquiring insulin samples or community support.  **Online Resource Links:** How to Get Health Insurance if You’re Unemployed (Diatribe), At the Pharmacy and Told Your Meds Aren't Covered? Here's What to Do Next (Beyond Type 1), Insulin Cost-Saving Resource Guide (Association of Diabetes Care and Education Specialists), Get Insulin (getinsulin.org) |
| 10. Finances and Mental Health | 3:21 | **Summary:** MR shares personal experiences and information about the mental burden of T1D. She discusses financial stress, diabetes burnout, anxiety, and depression.  **Online Resource Links:** Mental Health Resources for People with Type 1 Diabetes (Beyond Type 1), Diabetes Burnout (Beyond Type 1) |
