# Supplementary material for: Empowering emerging adults with type 1 diabetes: crafting a financial and health insurance toolkit through community-based participatory action research
Source: Res Involv Engagem. 2024 Jul 23;10:75. doi: 10.1186/s40900-024-00602-1 (PMC11265338; doi:10.1186/s40900-024-00602-1)
Supplement: Supplementary file 2 — Supplementary Material 2 [file 40900_2024_602_MOESM2_ESM.docx]

| **Section and topic** | **Item** | **Reported on page No** |
| --- | --- | --- |
| 1: Aim | Report the aim of PPI in the study | 10 |
| 2: Methods | Provide a clear description of the methods used for PPI in the study | 14-15 |
| 3: Study results | Outcomes—Report the results of PPI in the study, including both positive and negative outcomes | 16-21 |
| 4: Discussion and conclusions | Outcomes—Comment on the extent to which PPI influenced the study overall. Describe positive and negative effects. | 21-25 |
| 5: Reflections/critical perspective | Comment critically on the study, reflecting on the things that went well and those that did not, so others can learn from this experience. | 24 |

**GRIPP2 Short Form Checklist**
