# Supplementary material for: Empowering emerging adults with type 1 diabetes: crafting a financial and health insurance toolkit through community-based participatory action research
Source: Res Involv Engagem. 2024 Jul 23;10:75. doi: 10.1186/s40900-024-00602-1 (PMC11265338; doi:10.1186/s40900-024-00602-1)
Supplement: Supplementary file 3 — Supplementary Material 3 [file 40900_2024_602_MOESM3_ESM.docx]

**Appendix B. Overview of the Revised Toolkit**

| **Video Title** | **Length (minutes)** | **Description** | **Learning Outcomes**  **(After viewing these resources, I am able to…)** |
| --- | --- | --- | --- |
| Module 1 –  How to Adult: Health Insurance and Finances | 4:08 | **Summary:** Jennifer, Zhanna, and Thomas (emerging adults with type 1 diabetes), Krystle (The Diabetes Link staff), and Amanda (diabetes advocacy professional, emerging adult with type 1 diabetes) introduce some of the everyday life changes that occur during emerging adulthood and discuss the stressors of getting your first insurance plan, balancing health insurance and finances.    **Supplemental Resources Provided:** One-Pager of Qualifying Life Events for Insurance Enrollment (Developed by The Diabetes Link and CAB members) | *Identify* qualifying life events and when a support plan might be needed.  *Create* an action plan to utilize support programs or organizations that offer support with care and supplies to emerging adults with T1D. |
| Module 2 –  Key Insurance Lingo | 6:32 | **Summary:** Jennifer, Zhanna, Thomas (emerging adults with type 1 diabetes), and Amanda (diabetes advocacy professional, emerging adult with type 1 diabetes) define common health insurance terms.    **Supplemental Resources Provided:** Health Insurance Terms Glossary (Developed by The Diabetes Link, UH Research Team, and CAB members) | *Define* and *summarize* common health insurance terms, including premium, deductible, co-insurance, and copay.  *Explain* insurance process terms, including formulary/preferred prescription coverage, tiers, prior authorizations, appeals, and out-of-pocket maximums.  *Discuss* an explanation of benefits.  *Compose* key talking points to communicate with health insurance and providers about insurance concerns. |
| Module 3 –  Qualifying for Health Insurance | 5:04 | **Summary:** Jennifer, Zhanna, and Thomas (emerging adults with type 1 diabetes) and Amanda (diabetes advocacy professional, emerging adult with type 1 diabetes) explain their experiences starting on their own health insurance plans, introduce types of insurance plans, how to figure out what your new plan costs, how to figure out what your new plan covers (medication formulary), and how to plan for income changes or gaps in health insurance.  **Supplemental Resources Provided:** Choose Your Own Insurance Adventure—Figuring Out Which Insurance Plan Works for Your Situation (Developed by The Diabetes Link, UH Research Team, and CAB members) | *List* health insurance qualifications for employer-sponsored private health insurance plans and alternative health insurance plans.  *Determine* the enrollment period for my potential health insurance, including qualifying life events.  *Describe* the relationship between lapses in health insurance and dangerous diabetes self-management, such as rationing insulin and supplies.  *Construct* a plan to prevent lapses in health insurance. |
| Module 4 –  Choose a Plan and Enrolling | 5:46 | **Summary:** Destiny and Shay (emerging adults living with type 1 diabetes), Ebne (clinical diabetes pharmacist), and Christine (expert diabetes community advisor in medications, living with type 1 diabetes) discuss how to pick an insurance plan, what to consider when selecting a plan (deductible, out-of-pocket max), and describe how to use health maintenance organizations, preferred provider organizations, high-deductible health plans, and prescription drug coverage.  **Supplemental Resources Provided:** Health Insurance Plan Type Matrix, Questions to Ask When Enrolling in a New Insurance Plan, All About Deductibles One-Pager (Developed by The Diabetes Link and CAB members) | *Identify* what type of health insurance plan one has and its features (high-deductible, HSA, FSA, employer-sponsored, private, etc.).  *Recognize* differences among health insurance plans.  *Determine* which health insurance plan is best for one's individual needs based on benefits and premiums.  *Assess* options and *develop* a plan to maximize insurance benefits with health insurance providers and/or employers. |
| Module 5 –  The Power of Medicaid and Public Assistance | 7:54 | **Summary:** Destiny (emerging adult living with type 1 diabetes), Ebne (clinical diabetes pharmacist), Patti (Medicaid content expert), and Christine ( expert diabetes community advisor in medications, living with type 1 diabetes) define Medicaid, describe the benefits of Medicaid, different qualifying situations for Medicaid, Medicaid expansion, what is covered on Medicaid, and how to enroll, discuss provider bias and self-advocating for acquiring diabetes medications and technology, how to use Medicaid to navigate the healthcare system, and how to ensure you do not have lapses in Medicaid coverage.  **Supplemental Resources Provided:** Script of Questions to Ask Medicaid Providers, All About Medicaid One-Pager, Guide to Public Assistance Programs (Developed by The Diabetes Link and CAB members) | *Define* Medicaid and public assistance.  *Describe* the Medicaid enrollment process.  *Identify* where to access Medicaid and public assistance resources or support in my state.  *Determine* if I am eligible for and enroll in Medicaid/public assistance if applicable.  *Contact* Medicaid and public assistance representatives to *determine* (a) what benefits/resources I have the right to and (b) how to access and enroll in those benefits/resources.  *Locate* additional help when current representatives/ support does not meet my needs. |
| Module 6 –  Financial Planning and Comparing Insurance Costs | 10:29 | **Summary:** Shay (emerging adult living with type 1 diabetes), Ebne (clinical diabetes pharmacist), and Christine (expert diabetes community advisor in medications, living with type 1 diabetes) discuss considerations for budgeting your finances and health costs. Then, the viewers are walked through a health insurance cost worksheet with an example of an individual with diabetes costs.  **Supplemental Resources Provided:** Insurance Plan Comparison Tool (a fillable spreadsheet to compare different plans and costs), Sample Insurance Plan Comparison Tool (how to use the tool), How to Read Your Explanation of Benefits and Diabetes Annual Budget Worksheet (Developed by The Diabetes Link and CAB members) | *Identify* components of my insurance plan (in order to apply relevant pieces to my budget).  *Evaluate* my monthly/annual health care expenses.  *Determine* the percentage of my weekly/biweekly paycheck that will be saved for/contributed to healthcare expenses.  *Create* a healthcare expenditures budget and a plan to balance said budget on a monthly or yearly basis. |
| Module 7 –  You Have an Insurance Plan, Now What? | 3:50 | **Summary:** This is one of the original videos from the Diabetes Link Insurance 101 series. Stewart (health insurance expert living with type 2 diabetes), and Lissie, Naomi, Sam, and Shay (emerging adults living with type 1 diabetes) discuss how they use their health insurance plans to access in-network diabetes care, how to order diabetes durable medical supplies, how to order medications through mail-order pharmacies or commercial pharmacies, how to determine what your insurance covers and how to self-advocate when you speak with insurance companies.  **Supplemental Resources Provided:** N/A | *Examine* a formulary in order to *differentiate* costs between tiers.  *Describe* how formulary changes might impact existing prescriptions. *Create* a plan for getting necessary medications.  *Identify* strategies for communicating with insurance companies. |
| Module 8 –  HSA, FSA, What Does This Mean Anyway? | 5:57 | **Summary:** Nada, John, Shayna (emerging adults living with type 1 diabetes), and James (clinical pharmacist) describe Health Savings Accounts (HSA) and Flexible Spending Accounts (FSA), their benefits and limitations, their experiences utilizing them, how to use them to assist with budgeting, their tax benefits, and their HSA and FSA reimbursement and contribution rules.  **Supplemental Resources Provided:** HSA versus FSA Comparison Table (Developed by The Diabetes Link and CAB members) | *Differentiate* an HSA and FSA.  *Compare* HSAs and FSAs.  *Determine* whether you can get an HSA or FSA based on your plan and *determine* how to best utilize it.  *Explain* why health insurance plans that offer tax savings are important for emerging adults with diabetes. |
| Module 9 –  Cost-Saving Tips and Tricks | 8:18 | **Summary:** Sun and Deanelle (emerging adults living with type 1 diabetes), Stephanie (insurance content expert, living with type 1 diabetes), and game show host Paloma (social media influencer, emerging adult with type 1 diabetes) participate in a game show about cost-saving tricks to manage diabetes-related expenses. Game show points are awarded for each correct tip (verified by Stephanie) that Sun and Deanelle give. Cost-saving tips discussed include using diabetes wellness programs to save money on insurance premiums, navigating prescription formulary changes, filling prescriptions to save money (is 30 days or 90 days cheaper, which pharmacy is preferred by your insurance?), tips to reduce the cost of insulin (manufacturer coupons, patient assistance programs), how to utilize state copay caps, how to use state emergency laws for insulin and community support for health-related expenses.  **Supplemental Resources Provided:** Cost-Saving Cheat Sheet (Developed by The Diabetes Link and CAB members) | *List* common cost-saving techniques.    *Identify* which strategies and/or programs to utilize based on an individual's situation.    *Determine* what spending counts towards deductibles and out-of-pocket costs. |
| Module 10 –  Bridging Potential Gaps | 8:39 | **Summary:** Nada, John, Shayna (emerging adults living with type 1 diabetes), and James (clinical pharmacist) discuss previous experiences with personal gaps in health insurance coverage, when to anticipate gaps in coverage, how to plan ahead to try to avoid insurance coverage gaps and how to ensure you do not lose access to medication and diabetes supplies.  **Supplemental Resources Provided:** Pathways for Pharmacy Route and Pathways for Durable Medical Equipment (Developed by The Diabetes Link and CAB members) | *Determine* if my healthcare provider(s) are in network.  *Determine* if my insulin and supplies are covered by my insurance.  *Discuss* any potential changes to, or problems with, my prescription coverage with my insurance provider before I am due for a visit to my healthcare provider.    *Question* the process of obtaining a CGM/pump/other diabetes supplies to the appropriate individuals/entities.  *Differentiate* between a durable medical equipment company and a pharmacy. |
| Module 11 –  Challenges: Pause, Reflect, Don't Give Up! | 6:58 | **Summary:** Crystal, Steven, and Kaitlyn (emerging adults living with type 1 diabetes), Persis (diabetes psychologist, living with type 1 diabetes), Diana (clinical diabetes pharmacist), and Eugenia (diabetes care and education specialist, living with type 1 diabetes) discuss barriers to diabetes self-management and how to apply the problem-solving framework to remain calm and solve problems related to medication access and costs.  **Supplemental Resources Provided:** Guide to Self-Advocacy, Traveling with Diabetes, Natural Disaster/Emergency Preparedness, Navigating Insurance Stress/Anxiety (Developed by The Diabetes Link and CAB members) and Coupons and Cost Savings for Diabetes Technology and Coupons and Cost-Savings for Insulin (Developed by/for the Association of Diabetes Care and Education Specialists) | *Identify* potential challenges emerging adults with T1D may face in navigating the healthcare system or receiving care and potential problem-solving actions.  *Summarize* potential financial resources that may be utilized during problem-solving.  *Explain* how to self-advocate while solving problems related to navigating the healthcare system and receiving care.  *Devise* a plan that assists in staying calm and organized to promote problem-solving. |
| Module 12 –  Navigating the System and Communicating with the Healthcare Team | 8:02 | **Summary:** Crystal, Steven, and Kaitlyn (emerging adults living with type 1 diabetes), Persis (diabetes psychologist, living with type 1 diabetes), Diana (clinical diabetes pharmacist), and Eugenia (diabetes care and education specialist, living with type 1 diabetes) discuss how to communicate with your insurance provider and healthcare team to ensure continued coverage and access to diabetes technology and medications.  **Supplemental Resources Provided:** Health Insurance Provider Phone Call Preparation Checklist, Strategies for Preparing for Conversations with Insurance, and Health Insurance Provider Communication Log (Developed by The Diabetes Link and CAB members) | *Formulate* at least two ways to contact my healthcare and health insurance providers.    *Determine* the best way to address a quick question versus a lengthy comment/question/concern with my healthcare and health insurance providers.    *Explain* each medical product/service I need for my diabetes care.    *Communicate* my comments, questions, and concerns to relevant parties with confidence. |
| Module 13 –  The Emotional Side of Dealing with Insurance | 6:16 | **Summary:** Crystal, Steven, and Kaitlyn (emerging adults living with type 1 diabetes), Persis (diabetes psychologist, living with type 1 diabetes), Diana (clinical diabetes pharmacist), and Eugenia (diabetes care and education specialist, living with type 1 diabetes) discuss how stressful diabetes self-management, insurance, and healthcare costs are. They share personal stories and experiences and provide tips for getting support from the community, your family, peers, and the healthcare team, as well as how to advocate for yourself and use coping strategies and resources.  **Supplemental Resources Provided:** Talk Tracks: Example Exchanges for Navigating Lack of Support During Periods of Insurance-Related Emotional Stress and Support Programs and Resources (Developed by The Diabetes Link, UH Research Team, and CAB members) | *Describe* my current mental health state when experiencing financial and/or insurance-related stressors.    *Identify* who is on my emotional support team.    *Examine* ways to expand my diabetes community for support surrounding challenges with finances and/or insurance.    *Self-evaluate* an individual's mental health to determine the appropriate level of support needed and *list* pathways to seeking professional help. |
